# Brainstem Pain-Modulation Circuitry and Its Plasticity in Neuropathic Pain: Insights From Human Brain Imaging Investigations

**DOI:** 10.3389/fpain.2021.705345

**Published:** 2021-07-30

**Authors:** Emily P. Mills, Kevin A. Keay, Luke A. Henderson

**Affiliations:** Brain and Mind Centre, School of Medical Sciences (Neuroscience), University of Sydney, Sydney, NSW, Australia

**Keywords:** periaqueductal grey, rostral ventromedial medulla, locus coeruleus, subnucleus reticularis dorsalis, chronic neuropathic pain, conditioned pain modulation, functional magnetic resonance imaging, analgesia

## Abstract

Acute pain serves as a protective mechanism that alerts us to potential tissue damage and drives a behavioural response that removes us from danger. The neural circuitry critical for mounting this behavioural response is situated within the brainstem and is also crucial for producing analgesic and hyperalgesic responses. In particular, the periaqueductal grey, rostral ventromedial medulla, locus coeruleus and subnucleus reticularis dorsalis are important structures that directly or indirectly modulate nociceptive transmission at the primary nociceptive synapse. Substantial evidence from experimental animal studies suggests that plasticity within this system contributes to the initiation and/or maintenance of chronic neuropathic pain, and may even predispose individuals to developing chronic pain. Indeed, overwhelming evidence indicates that plasticity within this circuitry favours pro-nociception at the primary synapse in neuropathic pain conditions, a process that ultimately contributes to a hyperalgesic state. Although experimental animal investigations have been crucial in our understanding of the anatomy and function of the brainstem pain-modulation circuitry, it is vital to understand this system in acute and chronic pain states in humans so that more effective treatments can be developed. Recent functional MRI studies have identified a key role of this system during various analgesic and hyperalgesic responses including placebo analgesia, offset analgesia, attentional analgesia, conditioned pain modulation, central sensitisation and temporal summation. Moreover, recent MRI investigations have begun to explore brainstem pain-modulation circuitry plasticity in chronic neuropathic pain conditions and have identified altered grey matter volumes and functioning throughout the circuitry. Considering the findings from animal investigations, it is likely that these changes reflect a shift towards pro-nociception that ultimately contributes to the maintenance of neuropathic pain. The purpose of this review is to provide an overview of the human brain imaging investigations that have improved our understanding of the pain-modulation system in acute pain states and in neuropathic conditions. Our interpretation of the findings from these studies is often guided by the existing body of experimental animal literature, in addition to evidence from psychophysical investigations. Overall, understanding the plasticity of this system in human neuropathic pain conditions alongside the existing experimental animal literature will ultimately improve treatment options.

## Introduction

Acute pain serves as a protective mechanism that leads to a behavioural response aimed at removing the individual from potential tissue damage. These responses can involve simple withdrawal reflexes in addition to more complex behaviours, for example, mounting fight or flight responses. Whilst such behaviours are generally important for survival, it is known that acute pain can also be inhibited in certain situations. For instance, combat soldiers often display a remarkable attenuation of pain, with one report detailing that as many as three-quarters of badly wounded soldiers report little to no pain and do not require analgesic medications (1). Critically, the brain region that contains the fundamental circuitry for producing flight/fight behavioural responses to acute pain also produces a profound analgesia (2, 3). Indeed, it is thought that following the initial warning provided by noxious inputs, these inputs are subsequently dampened to allow for effective behavioural responses to the threatening situation. The circuitry responsible for this behavioural-coupled pain modulation lies within the brainstem and it is thought that analgesic responses associated with higher cognitive function, such as stress-induced or placebo analgesia, likely tap into this brainstem pain-modulating circuitry to regulate incoming noxious inputs.

In addition to playing an important role in acute pain modulation, it is thought that the function of brainstem endogenous pain modulating circuits are involved in the development of chronic pain. Whilst most acute pain resolves as the initial injury heals, in some individuals pain persists for months or even years after the injury has resolved. Chronic neuropathic pain, i.e., pain that results from injury to the nervous system, develops with surprising frequency even after “controlled injuries” such as following surgical procedures. For example, it is estimated that 30–50% of individuals develop chronic pain following limb amputation, 20–30% following breast surgery, 10% following either inguinal hernia repair or caesarean section and even up to 12% of individuals following endodontic therapy (4, 5). Chronic neuropathic pain poses a significant health and social challenge, largely because patients experience a range of behavioural comorbidities including depression, anxiety and sleep disturbances (6, 7). Current treatment regimens show limited effectiveness, for example, only 11% of individuals with painful trigeminal neuropathy respond to pharmacotherapy with a >50% reduction in pain intensity (8). Poor management of chronic pain is a key driver of the current opioid epidemic and highlights the fact that current treatments for chronic pain are inadequate and need to be greatly improved.

One of the limitations of treating chronic neuropathic pain conditions is the limited understanding of the mechanisms that generate and maintain persistent pain in some individuals following nerve injury. Whilst most neuropathic pain is initiated by damage to a peripheral nerve, the transition from the initial acute pain to a chronic pain state likely results from changes at, or above, the level of the primary nociceptive synapse at the dorsal horn (DH) or spinal trigeminal nucleus (SpV). Studies using preclinical models of neuropathic pain have reported that nerve injury evokes neuronal degeneration in the region of DH/SpV (9–13), which is thought to result from excessive afferent excitation (14, 15). In addition, both preclinical and human post-mortem studies have reported chronic glial activation in the DH/SpV associated with chronic neuropathic pain (16–19). These changes are observed alongside a myriad of alterations in higher brain centres and are critical for the persistent activation of ascending pain pathways, ultimately contributing to the constant perception of pain. It is still unclear why these events occur in some individuals and not others, even after seemingly identical injuries, and thus this remains the focus of much investigation.

One aspect being explored is the idea that dysfunction within the brainstem pain-modulation system contributes substantially to the changes within the DH/SpV following nerve injury. For example, multiple lines of research suggest that chronic neuropathic pain is associated with a shift in pain-modulation system functioning, such that the overall effect is the *facilitation* of pain and pain-related behaviours (20–22). Some investigations suggest that the state of the pain-modulation system prior to injury alters the propensity for an individual to develop chronic pain (23, 24). One method used to explore endogenous analgesic efficacy is conditioned pain modulation (CPM) ability, i.e., how effective is one noxious stimulus at reducing the perceived intensity of a second noxious stimulus (25). Reduced CPM ability is associated with increased postoperative pain (25), the presence of chronic pain conditions (26–28) and the effectiveness of analgesic medications (29). These findings strongly suggest that the effects of descending brain circuits on the DH/SpV are important for the development of chronic pain and that ongoing activity in this system may indeed predispose individuals to developing long-term pain and underlie analgesic treatment effectiveness. Indeed, in individuals whose pain resolves following nerve injury, effective descending control over the primary nociceptive synapse may limit the over-excitation from damaged nerves and protect the DH/SpV from undergoing the aforementioned neural and glial changes associated with persistent pain.

Until recently, it has been challenging to directly assess the structure and function of the brainstem pain-modulation system in acute and chronic pain processing in *humans*. However, advances in MRI scanner hardware and analysis techniques have recently allowed researchers to more directly examine small brainstem regions in humans (30–32). This has permitted the exploration of the function of this system in acute pain processing and during pain-modulation paradigms, and has allowed researchers to begin studying the structure and function of the endogenous pain-modulation circuitry in individuals with various chronic pain conditions, including chronic neuropathic pain. The purpose of the current review is to provide an overview of human brain imaging studies that have defined the endogenous pain-modulation circuitry in acute pain settings, and to highlight what is currently known about the function of these circuits in individuals with chronic neuropathic pain. Importantly, the review explores the results of these brain imaging investigations in the context of the existing experimental animal literature and clinical psychophysical studies.

## Brainstem Endogenous Pain-Modulation Circuitry

**Figure 1:**
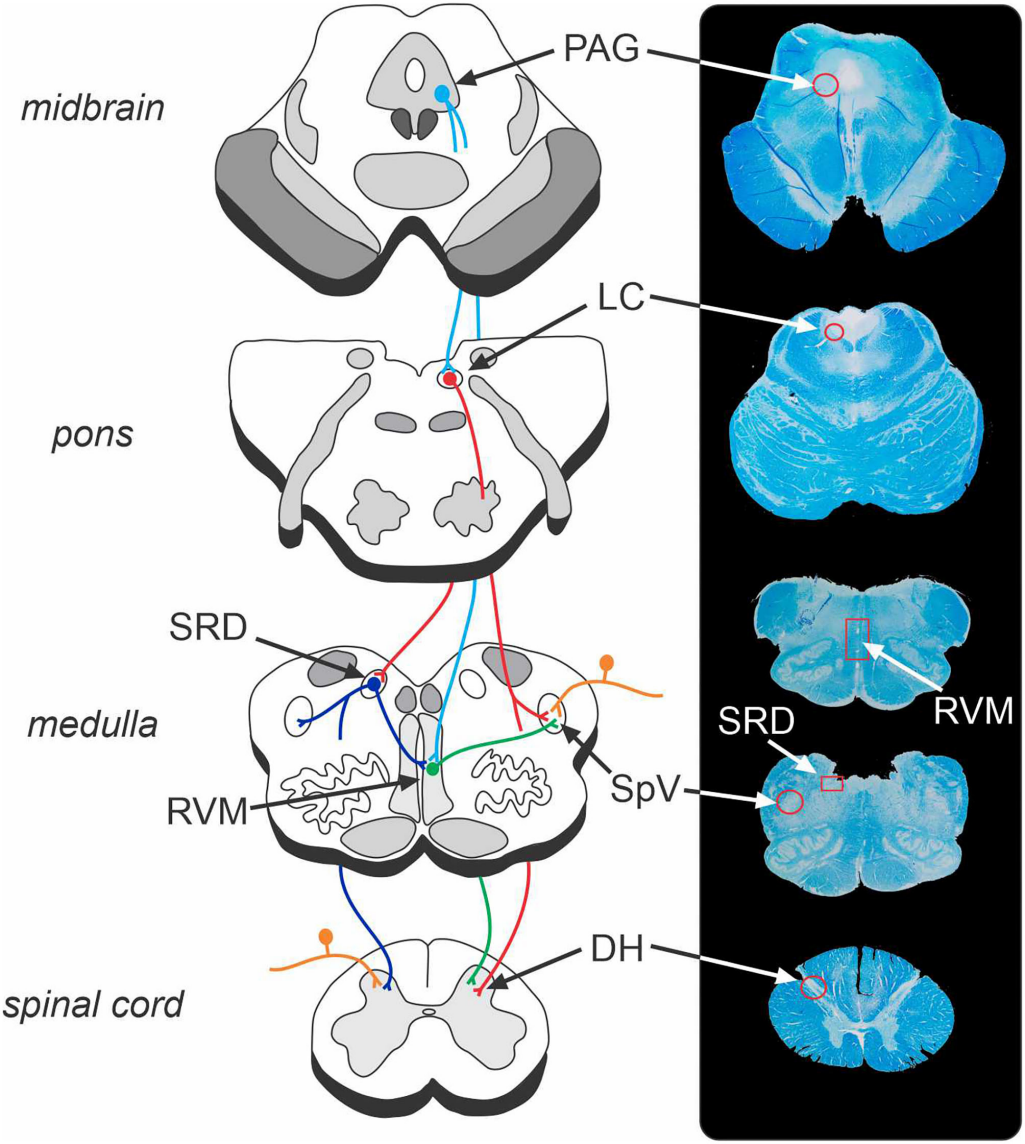
Brainstem endogenous pain-modulation pathways and their corresponding location on a human brainstem histological specimen. The periaqueductal grey (PAG) – rostral ventromedial medulla (RVM) – dorsal horn (DH)/spinal trigeminal nucleus (SpV) pathway is a key system that can produce both analgesia and hyperalgesia. The locus coeruleus (LC) and subnucleus reticularis dorsalis (SRD) likewise send direct projections to the DH/SpV to modulate nociception. The endogenous pain-modulation sites are highly interconnected (for instance, LC-SRD projections and SRD-RVM projections) and thus act collectively to exert dynamic anti- and pro-nociceptive control over the primary nociceptive synapse. Orange, nociceptor afferent projections to DH and SpV; light blue, efferent projections from PAG; red, efferent projections from LC; green, efferent projections from RVM; dark blue, efferent projections from SRD. Note that corresponding structures exist bilaterally.

The brainstem contains a series of regions that receive ascending nociceptive information and likewise directly and indirectly regulate neurotransmission at the primary nociceptive synapse (33, 34). The net influence of these descending brainstem pathways is to inhibit and/or facilitate the primary synapse which modulates the pain experience by down- or up-regulating ascending neurotransmission (33, 35). Given the limited ability for human studies to explore the discrete and small nuclei located in the brainstem, the vast majority of studies investigating brainstem pain modulatory circuits have been conducted in experimental animals, primarily rodents (34, 35). As illustrated in Figure 1, these preclinical studies have identified several key brainstem structures that modulate pain, including the periaqueductal grey matter (PAG), rostral ventromedial medulla (RVM), locus coeruleus, and subnucleus reticularis dorsalis (SRD). The PAG-RVM-DH/SpV pathway is the most extensively studied and is thought to form the common final pathway for analgesic responses in various situations including as part of the behavioural responses to acute noxious stimuli. In addition, the locus coeruleus and SRD directly project to the DH/SpV to modulate neurotransmission (36–39), and the SRD has been heavily implicated in the CPM response (40, 41). Whilst these circuits are often investigated in a semi-independent manner, the various sites are likely to act collectively to exert dynamic anti-nociceptive and pro-nociceptive control over the DH/SpV, contributing to analgesia and hyperalgesia, respectively (22, 37, 42).

Brain imaging, particularly functional magnetic resonance imaging (fMRI), has allowed researchers to begin exploring the brain circuitry responsible for various types of analgesic and hyperalgesic responses in humans. Given the limited spatial resolution of most fMRI studies, for the most part these investigations have explored cortical sites associated with pain modulatory responses (43, 44). However, with increases in MRI field strengths and improvements in various analytical techniques, it has become feasible to explore the brainstem circuitry involved in pain modulation. For instance, researchers have employed analysis techniques such as specialised brainstem and cerebellum isolation procedures, including the spatially unbiased infratentorial toolbox (SUIT) (45), the creation of individualised mask regions (30), and removal of cardiovascular and respiratory signals using dedicated toolboxes such as DRIFTER (46) to study brainstem function. We and others (47) have begun to use the increased spatial resolution afforded by ultra-high field 7 Tesla MRI to resolve discrete brainstem regions involved in pain modulation, including individual columns of the PAG, to better elucidate their roles in processing and modulating pain.

### PAG-RVM-DH/SpV Pathway

**Figure 2:**
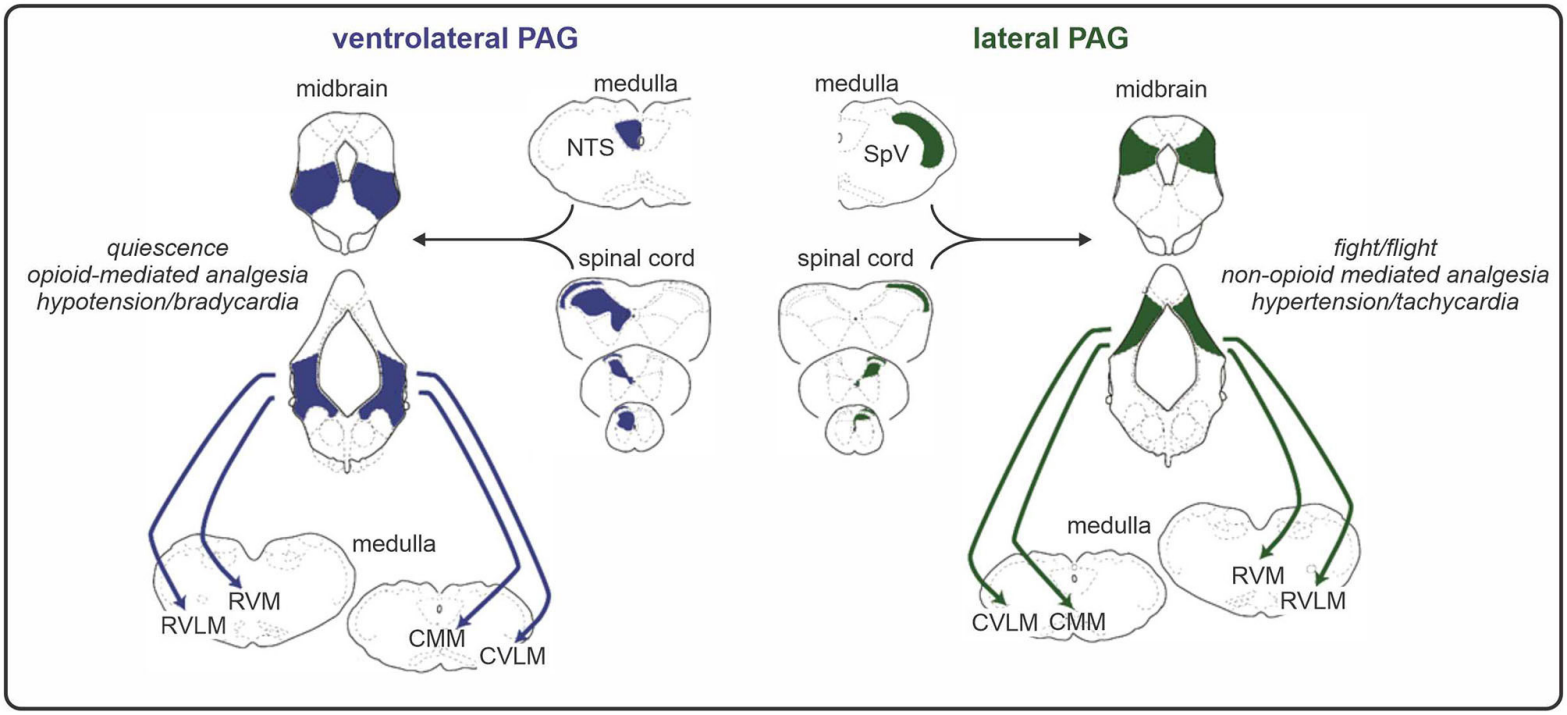
Functional and anatomical organisation of the midbrain periaqueductal grey (PAG). The ventrolateral column of the PAG receives direct input from the spinal cord dorsal horn and evokes an opioid-mediated analgesia alongside a distinct set of coping behaviours including quiescence. Conversely, the lateral PAG receives direct inputs from the dorsal horn/spinal trigeminal nucleus (SpV) that are organised in a somatotopic manner and can elicit fight or flight responses and a non-opioid mediated analgesia via its projections to medullary nuclei. RVLM, rostral ventrolateral medulla; RVM, rostral ventromedial medulla; CMM, caudal medial medulla; CVLM, caudal ventrolateral medulla; NTS, nucleus of the solitary tract.

Preclinical studies have shown that the PAG consists of longitudinal columns of grey matter that run rostro-caudally along the midbrain aqueduct. As illustrated in Figure 2, the columns of the PAG play distinct roles in analgesia and pain-related behaviours. Experimental animal investigations show that the ventrolateral column of the PAG (vlPAG) is involved in opioid-mediated analgesia, since morphine microinjection into this region produces a reduction in sensory pain behaviours across a range of analgesiometric tests (48). Neuronal excitation within vlPAG also produces a distinct set of behaviours including hyporeactivity and quiescence (49). In contrast, activation of the lateral and dorsolateral PAG columns evokes a non-opiate mediated analgesia that is often coupled to the behavioural responses of fight/flight (49, 50). The lateral PAG receives direct inputs from the DH and SpV organised in a crude somatotopic map, with orofacial afferents terminating rostrally and afferents from the legs terminating caudally (51). This somatotopic organisation is also consistent with the behavioural response when activated with rostral lateral PAG stimulation evoking forward defence and caudal lateral PAG stimulation evoking a fleeing response (52). Given this specificity, it is likely that lateral PAG-evoked analgesic responses are also somatotopically organised.

Neuroanatomical tract tracing studies have shown that the PAG sends minimal direct projections to the DH/SpV and instead indirectly exerts its effects via projections to the RVM (35, 53). The RVM is defined functionally as the midline pontomedullary area in which opioid microinjection produces analgesia (54), and consists of several nuclei including the midline nucleus raphe magnus (NRM) and lateral nucleus reticularis gigantocellularis (NGc). RVM neurons project directly to the DH laminae that receive nociceptor primary afferents via the dorsolateral funiculus (33, 55, 56) and to the SpV (57–60). Together, the PAG-RVM-DH/SpV axis can have an inhibitory effect on nociceptive transmission, since electrical stimulation of either region can suppress nociceptive neurons within the DH (61) and SpV (60, 62, 63), and can inhibit nocifensive responses (33, 64). The analgesic effects of directly stimulating the PAG have likewise been shown in humans (65), and direct PAG stimulation is sometimes used to reduce the intensity of pain in individuals with various chronic pain conditions (66).

Whilst much focus has been on the ability of this brainstem pathway to reduce pain, excitation of the PAG-RVM system can have a facilitating effect on nociceptive transmission at both the DH (38, 67) and SpV (68). For instance, electrical stimulation within the RVM can produce biphasic effects-stimulation at relatively high current intensities can inhibit DH neurons, whereas RVM stimulation at lower intensities can facilitate DH neurons (69). It is now recognised that within both the PAG and RVM, there are scattered populations of neurons that have distinct nociceptive-modulating properties (55, 70). These populations include Off-cells, which show a pause in their firing immediately before a nocifensive behaviour and are thus considered anti-nociceptive (71, 72), and On-cells which fire during a pain behaviour and are therefore classified as pro-nociceptive (72). Accordingly, nociceptive threshold varies with the balance between the activity of On- and Off- cell populations, that is, pain behaviours are greater when On-cells synchronously enter a period of activity and Off-cells a period of silence (72).

Although most of our basic understanding of the PAG-RVM-DH/SpV pathway stems from experimental animal investigations, investigators have begun to report changes in human brainstem and spinal cord fMRI activation patterns associated with pain and pain modulation. Relatively early fMRI studies identified that somatic and visceral noxious stimulation leads to activation of brainstem regions including the PAG and RVM (73). Other studies have identified activation at the primary nociceptive synapse, either at the DH or SpV, in addition to the PAG and RVM during noxious muscle and cutaneous inputs (74–79). The most compelling investigations have identified an association between the activation in, or signal coupling between, these regions and the intensity of pain reported during acute noxious inputs. For instance, a recent investigation employed a structural equation modelling approach to identify that the strength of directed fMRI signal coupling from the PAG to both the NRM and NGc within the RVM correlates with pain intensity ratings during noxious thermal stimulation (79). Interestingly, this investigation also identified that the BOLD signals within the PAG and NRM significantly correlate even at baseline, suggesting a tonic connectivity between these regions even prior to noxious stimulation. In another recent fMRI investigation, a novel MRI acquisition protocol was used to explore the activation of the spinal cord in addition to higher cortical and subcortical areas under conditions of high and low noxious thermal stimulation (76). The authors identified that under conditions of high > low intensity stimuli, there is enhanced activation in the DH ipsilateral to the stimulus, in addition to the midbrain in the area of PAG. Furthermore, the investigators found that the strength of PAG-DH fMRI signal coupling (“functional connectivity”) predicts individual pain intensity ratings to the noxious thermal stimuli. Whilst direction cannot be inferred using functional connectivity analyses, the strong positive correlation between PAG-spinal cord coupling and pain intensity ratings likely reflects the descending control of the PAG over the DH during acute noxious stimulation (76).

In addition to the investigations into the PAG-RVM-DH system during acute pain processing, brain imaging studies have begun to explore brainstem and spinal cord functioning during experimental pain-modulation paradigms and under pharmacological manipulation of the analgesic system. These investigations have complimented the existing experimental animal literature in that they have revealed changes in brainstem activity during analgesic responses. For instance, an earlier study identified that acupuncture produces significant de-activations in the caudal vlPAG and NRM, and significant activations within the rostral vlPAG (80), suggesting that this type of analgesia is associated with widespread and complex changes in PAG-RVM system activity. Furthermore, the authors found that verum acupuncture produces significantly greater activations in the vlPAG than sham acupuncture, possibly supporting a role for endogenous opioids in verum acupuncture analgesia (81). Several fMRI investigations have also reported on the contribution of the PAG-RVM-DH system to the offset analgesia phenomenon, whereby there is a disproportionate reduction in perceived pain following a small temperature decrease during noxious thermal stimulation (82, 83). In these studies of offset analgesia, a frequently-used paradigm involves applying a noxious thermal stimulus, and then raising the temperature by 1–3 degrees Celsius before lowering it to the original noxious temperature. Often, there is a disproportionate decrease in the pain intensity reported following the return to the original temperature (82–84). In their investigation, Yelle et al. identified that offset analgesia is associated with an increase in activity in the PAG and in medullary regions including the NRM and NGc (82). In contrast, Sprenger et al. identified significantly *reduced* BOLD responses in the dorsal spinal cord under conditions of offset analgesia compared to when a constant stimulus was applied. Importantly, the time course of the BOLD response followed the time-course of perceived pain ratings, and not the temperature of the stimulus (83). Together, these investigations suggest that this type of analgesia involves an enhanced activation of the PAG and RVM, which in turn results in reduced nociceptive processing at the level of the spinal cord.

Human brain imaging investigations have also implicated the PAG-RVM-DH system in pain modulation by cognitive factors, such as attending to pain and expectations surrounding pain relief. A recent study into the attentional modulation of pain found that PAG activity is greater during pain under difficult vs. easy cognitive task conditions, and that the magnitude of attentional analgesia is correlated with RVM activity (30), the results of which have been recently confirmed in a follow-up investigation with a greater number of subjects (85). In this later study, a psycho-physiological interactions analysis also revealed a greater PAG-RVM task-based functional connectivity under pain conditions during harder vs. easy cognitive tasks, providing further support for the role of this system in attentional analgesia. In addition, a series of investigations exploring placebo analgesia found that pain-relief achieved by a placebo cream is associated with increased activity in a region identified as the PAG and in the medulla/pons near the RVM (86), and is also associated with decreased activity in the DH (87). It is likely that these signal changes underlie opioid-mediated placebo analgesia driven by the PAG-RVM-DH/SpV pathway, since the administration of the opioid antagonist naloxone can reduce activity in this pathway and diminish the magnitude of pain-relief achieved by a placebo cream (86).

In support of this hypothesis, another recent study pharmacologically manipulated the opioid system during fMRI and revealed activity changes throughout the PAG-RVM-DH neuroaxis (78). Specifically, this investigation explored the phenomenon of paradoxical hyperalgesia following the abrupt discontinuation of short-term opioid infusion. Following remifentanil discontinuation, there was greater noxious stimulus-evoked activation within the PAG and RVM compared with a saline control, and the authors used a multivoxel pattern analysis to identify an effect of opioid cessation at the DH during noxious thermal processing (78). Alongside the study by Eippert and colleagues, this investigation provides strong evidence for the role of the PAG-RVM-DH/SpV pathway in opioid-mediated analgesia in humans, and together these findings complement a wealth of existing experimental animal data into the opioid analgesic system.

Recent investigations have also provided evidence for a role of the PAG-RVM-DH/SpV circuitry in hyperalgesia in humans. An early fMRI investigation explored the supra-spinal contributions to central sensitisation, that is, enhanced sensitivity at the primary nociceptive synapse that contributes to secondary hyperalgesia (88). The authors identified increased activations in the PAG during non-noxious punctate stimulation of a secondary hyperalgesic area compared to a control site, implicating this region in the development of centrally-mediated hyperalgesia. A more recent investigation combined simultaneous brainstem and spinal cord fMRI to examine the supra-spinal control over the DH during a temporal summation paradigm, whereby repeated moderate noxious stimuli delivered at high frequencies results in increasing pain (89). The authors found that temporal summation was associated with significantly greater activation in the lateral region of the PAG, the RVM and also in the area encompassing the DH. Finally, a recent study into nocebo hyperalgesia used a cortico-spinal imaging approach to identify that fMRI signal coupling strength between the lateral PAG and the spinal cord correlated with nocebo response magnitude (90), suggesting that negative expectations surrounding pain can lead to hyperalgesia via PAG-driven pro-nociception at the DH.

Overall, these fMRI studies have begun to elucidate the role of the PAG-RVM-DH/SpV circuitry in pain processing and modulation in humans, and further advances in MRI scanner hardware and analysis techniques will permit a more detailed exploration of the discrete nuclei and subregions involved in several of these processes.

### Locus Coeruleus and Subnucleus Reticularis Dorsalis

In addition to the PAG-RVM-DH/SpV pain modulatory axis, it is well-known from experimental animal studies that other brainstem regions also have the ability to significantly modulate incoming noxious information. One such region, the locus coeruleus, is located in the dorsal pons and is a major source of noradrenaline in the brain along with nearby noradrenergic cell groups (91). It has been shown that noradrenaline released by the locus coeruleus can mediate arousal and stress responses (92, 93), and can have both inhibitory and facilitating effects on neurotransmission throughout the central nervous system via the activation of alpha-1 and alpha-2 adrenoreceptors (91, 94). During acute noxious stimulation, descending locus coeruleus noradrenergic neurons target the DH/SpV directly where they can inhibit nociceptive neurotransmission (95, 96).

As well as direct projections, the locus coeruleus can modulate pain *via* its noradrenergic projections to other brainstem regions, including the subnucleus reticularis dorsalis (SRD) in the medulla (97, 98), and also shows reciprocal projections with the medial prefrontal cortex (mPFC) (99, 100). Locus coeruleus neuron terminals within the SRD appear to be pro-nociceptive, since noradrenaline activates alpha-1 adrenoreceptors on SRD neurons which facilitate pro-nociceptive output from the SRD (101). The locus coeruleus and noradrenergic system in general are likewise involved in acute pain processing and modulation in humans. Intrathecal administration of the alpha-2 adrenergic agonist clonidine reduces capsaicin-induced pain and hyperalgesia in healthy individuals (102).

More recently, fMRI studies have explored the role of the locus coeruleus in pain and pain-modulation. A recent investigation used a structural equation modelling approach to identify that fMRI signal coupling between the locus coeruleus and the NRM within the RVM *prior* to the application of noxious stimuli significantly correlates with pain intensity ratings during stimulation (79). Interestingly, the authors identified that this correlation with pain intensity ratings is no longer significant *during* the noxious stimulation, despite the locus coeruleus maintaining significant connectivity strengths with both the NRM and NGc before, during, and after stimulation on average across participants (79). This may reflect a shift in the role of the locus coeruleus throughout the pain experience, and suggests that the interaction between this region and other pain-modulation circuits during the anticipation of pain can directly influence the individual's experience of noxious stimuli. Contrastingly, fMRI activation studies have identified changes in activity within the dorsolateral pons in the area of locus coeruleus *during* acute noxious stimulation (73, 75). Khan and Stroman (75) found that activity in both the locus coeruleus and DH inversely correlates with inter-individual pain intensity ratings during noxious thermal stimulation, possibly reflecting locus coeruleus-driven anti-nociception at the DH under lower pain intensities. Several studies have also begun to explore the role of the locus coeruleus in modulating pain during pain-modulation paradigms. It has been reported that acupuncture analgesia produces de-activations in the locus coeruleus (80), and placebo analgesia is associated with activation in an area consistent with the anatomical location of locus coeruleus, extending into the outer margins of the PAG (86). Furthermore, attending away from thermal pain during a cognitively demanding task has also been associated with locus coeruleus signal intensity changes (30). The authors also identified that within the locus coeruleus there is an interaction effect between the performance of the task and the temperature of the noxious stimulus, suggesting that this region plays a role in the attentional modulation of pain in humans (30).

The SRD, in addition to receiving noradrenergic input from the locus coeruleus, is able to modulate nociceptive transmission via direct projections with the DH/SpV (39, 103, 104). The SRD runs along the dorsal medulla and experimental animal studies have shown that it can play a pro-nociceptive role, since blocking SRD activity can reduce wide-dynamic-range (WDR) DH neuronal activity and inhibit pain behaviours during a single noxious stimulus (97, 105). Human acute pain studies have shown that the SRD is activated by noxious thermal stimuli (77), and also receives communication from the PAG during and after noxious thermal stimulation (79). In addition to its involvement in pain modulation during a single stimulus, the SRD plays a critical role in a phenomenon referred to as “diffuse noxious inhibitory controls” (DNIC) (37). The DNIC response involves a reduction in the pain intensity evoked by a single noxious stimulus by the administration of a second, remote noxious stimulus (106). That is, pain inhibits pain. DNIC is a powerful analgesic mechanism which can block transmission of nociceptive information by completely inhibiting WDR neurons in the deep DH and SpV (40, 106, 107). The DNIC effect is mediated by supraspinal sites that send descending projections to regulate neurotransmission at the deep DH/SpV. A series of investigations revealed that the SRD is the key region responsible for producing this analgesia, since lesioning this region significantly attenuates analgesia during dual noxious stimuli (41, 108).

**Figure 3:**
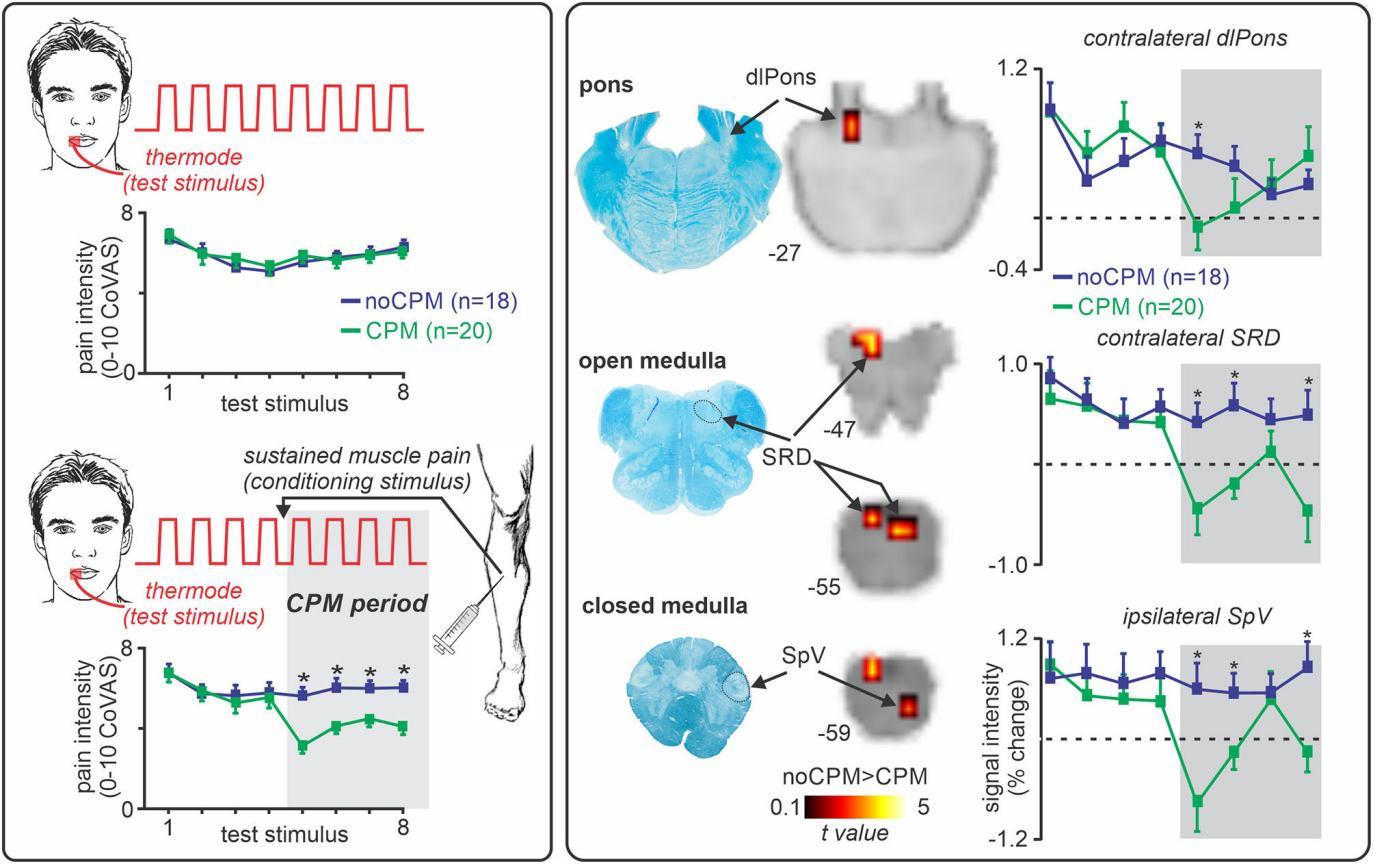
Role of the subnucleus reticularis dorsalis (SRD) in conditioned pain modulation (CPM). Left panel: CPM is assessed by measuring the change in pain intensity to a single noxious stimulus (“test stimulus,” noxious thermal stimulus), by the application of a second noxious stimulus (“conditioning stimulus,” sustained muscle pain). Approximately 50% of individuals experience a reduction in pain intensity during the conditioning stimulus (CPM responders, green), where the remainder do not (CPM non-responders, blue). *significant between-groups difference, two-sample *t*-test, *p* < 0.05. Right panel: During a CPM paradigm, CPM responders show a decrease in signal intensity changes in the dorsolateral (dl) pons, the SRD, and spinal trigeminal nucleus (SpV) compared to non-responders. *significant between-groups difference, two-sample *t*-test, *p* < 0.05. Figure modified with permission from Youssef et al. (77).

In humans, the DNIC phenomenon is termed “conditioned pain modulation” (CPM), and is considered a reliable test of endogenous analgesic capabilities. CPM is assessed by measuring the change in pain ratings to the first “test” stimulus by the application of a second “conditioning” stimulus. Whilst cortical processes may modulate CPM (109), the analgesia associated with this response relies on brainstem structures since CPM is absent in patients with complete spinal cord transection (110) and in patients with lateral medullary lesions (111). Recently, a functional brain imaging investigation in our laboratory explored the brainstem mechanisms underlying CPM in healthy humans (77) (Figure 3). We applied a series of noxious thermal stimuli to the lips and assessed the change in pain intensity response to these stimuli during the application of sustained tibialis anterior muscle pain. As Figure 3 illustrates, individuals who experienced a robust CPM analgesia showed reduced signal intensity changes in the SRD and SpV compared to those who did not (77), supporting the idea that the SRD inhibits the SpV during dual noxious stimulus processing. Whilst our investigation did not find significant CPM-related changes in the area of the PAG or RVM, there is evidence that the PAG is associated with CPM in humans (112, 113), and that the RVM is associated with the DNIC response in experimental animals (114, 115). This suggests that the mechanism underlying CPM analgesia involves a complex interaction between the SRD, PAG and RVM.

Many of the pain modulatory responses described above involve experimental paradigms that require higher cognitive appraisal, particularly in humans. It is therefore likely that descending inputs from areas such as the prefrontal and cingulate cortices are involved in modulating various brainstem sites such as the PAG. Indeed, such descending projections are involved in mediating analgesia associated with directing attention towards or away from a noxious stimulus (116), and anticipating pain (44). Experimental animal and human tract-tracing studies have shown afferent projections to the PAG from higher regions, including substantial inputs from the prefrontal cortex (117–119) and hypothalamus (117, 120). Additionally, resting state fMRI studies have shown ongoing signal coupling (“functional connectivity”) between the vlPAG and the anterior cingulate cortex (ACC) in healthy individuals (121, 122), and activation studies have revealed that the dorsolateral prefrontal cortex (dlPFC) can engage the brainstem pain-modulation circuitry to modulate pain under various circumstances (44, 109). For instance, in our CPM investigation, we found that those individuals who did *not* experience CPM analgesia showed enhanced functional connectivity between the SRD and dlPFC, suggesting that the dlPFC can inhibit the brainstem circuitry critical for CPM expression (109). In contrast, placebo analgesia is associated with increased activity within the dlPFC (44, 86), which positively correlates with placebo-induced activity changes within the PAG (44) and likely mediates this type of analgesia.

## Alterations in Brainstem Endogenous Pain-Modulation Circuitry in Chronic Neuropathic Pain

It is clear that the brainstem contains a network of descending circuits that modulate noxious information entering the central nervous system. This tonically active descending input can modulate incoming noxious drive as well as the on-going background activity of the DH/SpV which itself may elicit pain without an increase in peripheral nociceptor drive. Indeed, there is growing evidence from experimental animal studies that chronic neuropathic pain is associated with neuroplasticity within the brainstem pain-modulation system (20, 54). These cellular and neurochemical changes lead to a shift in the overall output of the DH/SpV, and this shift contributes to the initiation and maintenance of long-term neuropathic pain behaviours, including hypersensitivity to noxious and non-noxious stimuli (21, 101, 123), and complex behavioural disturbances (124, 125).

Human psychophysical studies have also indicated that chronic neuropathic pain is associated with a dysfunction of the endogenous pain-modulation system. For example, individuals with neuropathic pain demonstrate widespread alterations in the processing of both noxious and non-noxious stimuli at the site of injury and across the entire body (126). This can include enhanced pain in response to a noxious stimulus at the site of injury and surrounding body regions, i.e., primary and secondary hyperalgesia, as well as abnormal pain response to a non-noxious stimulus at the injury site or surrounding regions, i.e., primary and secondary allodynia. These characteristics suggest that neuropathic pain is associated with an overall up-regulation of sensory pain processing across the body, an effect that is partly mediated by the descending pain-modulation system (54, 127). In addition, neuropathic pain is often associated with a reduction in CPM ability, although it must be noted that the evidence is mixed and depends on the modality and location of test and conditioning stimuli [see detailed review by (128)]. When test and conditioning stimuli are delivered to non-affected body sites, individuals with painful trigeminal neuropathy (PTN) or painful chemotherapy-induced neuropathy show less efficient CPM compared to pain-free controls (28, 129). Furthermore, when the patients' ongoing clinical pain is considered the test stimulus, the application of a conditioning stimulus does not lead to a significant reduction in clinical pain intensity in patients with either peripheral neuropathic pain (130) or central post-stroke pain (131). These CPM investigations provide insight into the disrupted functioning of the endogenous pain-modulation system in various neuropathic pain conditions.

Research has also focused on the use of CPM as a potential diagnostic tool to predict chronic neuropathic pain development, determine the effectiveness of analgesic medications and predict the clinical manifestations of pain (24, 29, 132, 133). Indeed, there is evidence that CPM deficiencies precede the development of chronic pain and may indeed act as a risk factor for chronic pain development (24, 132). Reduced pre-operative CPM ability has been associated with the presence of chronic pain following thoracotomy surgery (24), and major abdominal surgery (132). However, it must be noted that a recent systematic review identified conflicting evidence for the ability of CPM to predict the clinical manifestations of neuropathic pain, such as pain intensity and duration, with 57% of included studies finding significant correlations (134). This may be due to the heterogeneity of CPM protocols employed in many of these studies.

There is also evidence that the pro-nociceptive component of the endogenous pain-modulation system is compromised in neuropathic pain states. For example, patients with chemotherapy-induced neuropathic pain experience enhanced pain responses during temporal summation paradigms (28), an effect thought to be in part mediated by descending brain circuits (89, 135, 136). Furthermore, the magnitude of pre-treatment temporal summation predicted the pain-relief achieved by ketamine in patients with refractory neuropathic pain (136), suggesting that an individual's pro-nociceptive capacity is related to the efficacy of certain analgesic medications. Although these investigations indicate that chronic neuropathic pain is associated with altered pain-modulation capabilities, psychophysical testing does not directly assess the intrinsic functioning of the pain-modulation regions and pathways. As such, extensive experimental animal investigations and, more recently, human brain imaging studies have begun to explore the nature of brainstem pain-modulation system functioning in states of ongoing pain following nerve injury, as detailed in the following sections.

### PAG-RVM-DH/SpV Plasticity

Evidence from human imaging and experimental animal studies strongly suggests that the changes along the PAG-RVM-DH/SpV pathways contribute to a shift towards a pro-nociceptive state, ultimately facilitating neurotransmission and promoting pain (22, 32). In addition to changes at the level of the DH/SpV [see (137) for recent review], studies using preclinical models of neuropathic pain have shown changes in vlPAG neuronal excitability and both spontaneous and stimulus-evoked firing patterns associated with pain behaviours (138–140). In addition, there are reports that neuropathic pain is associated with persistent glial activation, in particular astrogliosis in the PAG (124) and in the RVM (141, 142). Several investigations have identified changes in the firing properties of RVM On- and Off-cells following nerve injury (143–145). Both On- and Off- cells show lowered thresholds and altered responsivity to mechanical and thermal stimuli in a stimulus intensity graded manner (143). There are also reports that the spontaneous discharge rates of inhibitory RVM Off-cells are reduced (144, 145) and On-cell firing rates are increased in models of neuropathic pain (145) and deactivation of either the PAG or RVM or disrupting the RVM-DH pathway can reduce allodynia and hyperalgesia (21, 123). Together, these preclinical studies indicate that chronic neuropathic pain is associated with reduced descending tonic inhibitory and increased facilitatory drive from the RVM following nerve injury. Furthermore, there is evidence that the state of the PAG-RVM-DH/SpV pathways prior to nerve injury can influence the propensity for developing chronic pain behaviours. For instance, the selective disruption of RVM cells expressing μ-opioid receptors prior to nerve injury can protect against the development of pain behaviours including hypersensitivity to somatosensory stimuli (23). Furthermore, the authors report that the loss of RVM μ opioid receptor-expressing cells can reverse experimental neuropathic pain after its development, suggesting that brainstem function plays a critical role in both the development and maintenance of neuropathic pain behaviours.

**Figure 4:**
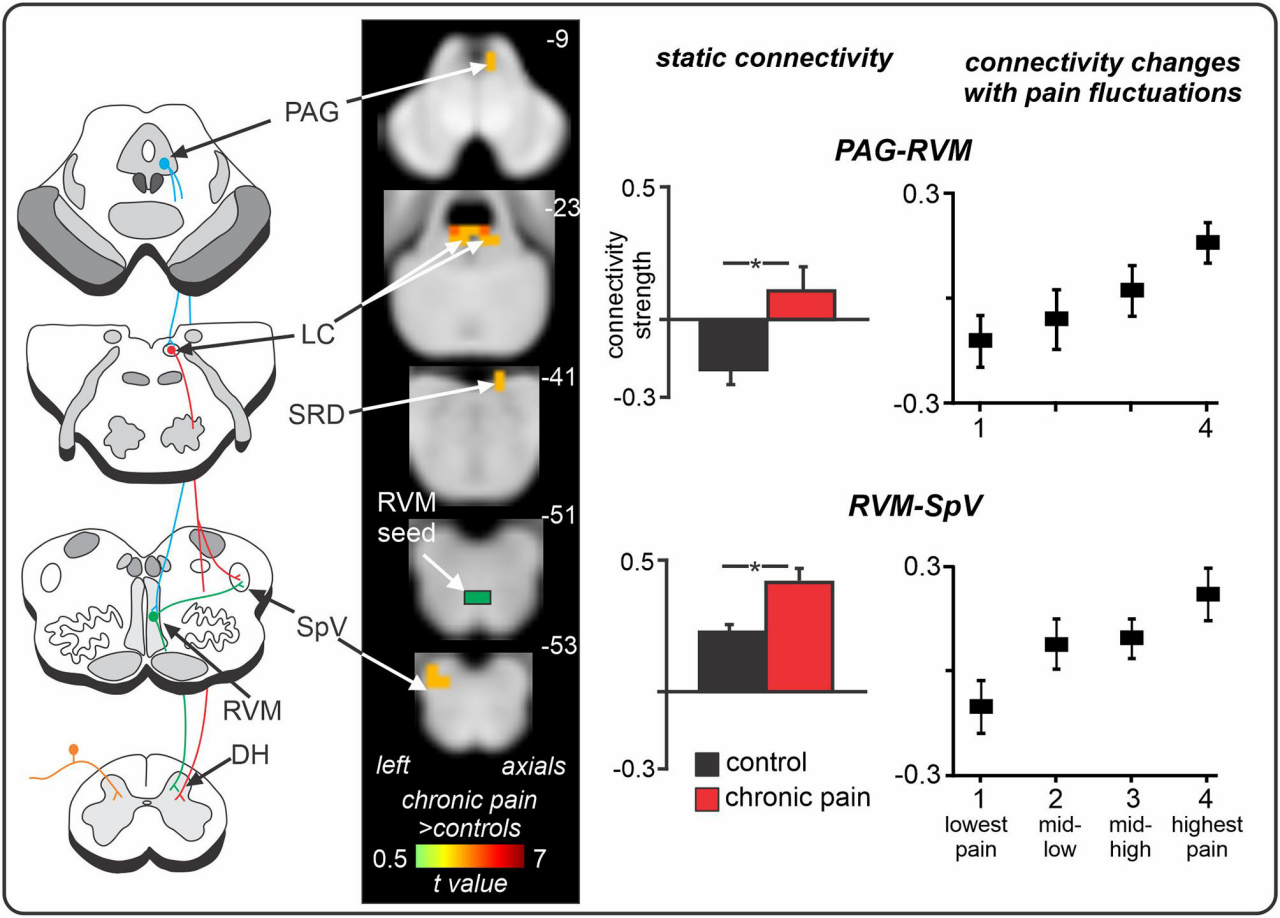
Spontaneous changes in periaqueductal grey (PAG) - rostral ventromedial medulla (RVM) - spinal trigeminal nucleus (SpV) fMRI signal coupling in chronic neuropathic orofacial pain. Compared to controls, individuals with chronic pain show enhanced positive functional connectivity between the RVM “seed” and the PAG and SpV, in addition to the locus coeruleus (LC) and subnucleus reticularis dorsalis (SRD). Furthermore, in neuropathic pain patients, as the intensity of their clinical pain changes throughout a 12-min fMRI scan, so too do their RVM connectivity strengths with the PAG and SpV. That is, when pain intensity is spontaneously low, RVM connectivity strengths with both the PAG and SpV are low; and when pain intensity is spontaneously high, RVM connectivity strengths are high and positive. *significant between-groups difference determined in a voxel-by-voxel analysis. Figure modified with permission from (32) and Mills et al. Journal of Pain Research 2020:13:2223–2235; Originally published by and used with permission from Dove Medical Press Ltd. (150).

Recent human brain imaging investigations have also begun to explore the PAG-RVM-DH/SpV axis in individuals with chronic neuropathic pain. Volumetric studies have revealed that trigeminal neuralgia is associated with increased grey matter volume in the PAG (146, 147), which may itself reflect glial activation or alternatively, changes in dendritic spines and synapses (148, 149). In a recent series of fMRI studies, we explored resting activity patterns in the PAG-RVM-SpV pathway in chronic orofacial neuropathic pain. Specifically, we studied the fMRI signal coupling (functional connectivity) between the RVM (a “seed” region of interest) and other brainstem regions, and found that positive functional connectivity over the entire scan (“static” connectivity) between the RVM and both the PAG and SpV was significantly enhanced in chronic neuropathic pain (Figure 4) (32). Considering the results from preclinical investigations, we suggest that this enhanced PAG-RVM-SpV functional connectivity in individuals with chronic orofacial neuropathic pain reflects a shift towards pro-nociception that ultimately contributes to the maintenance of ongoing pain. Given the evidence that RVM Off-cells show reduced spontaneous activity and On-cells show enhanced spontaneous activity following nerve injury (144, 145), it is conceivable that the enhanced signal coupling in the PAG-RVM-SpV pathway is driven predominantly by On-cells. In a subsequent investigation, we also found that moment-to-moment changes in PAG-RVM-SpV signal coupling matched spontaneous changes in pain intensity in individuals with chronic orofacial neuropathic pain (150). That is, when an individual's pain was spontaneously high, so too were RVM connectivity strengths with the PAG and SpV, and vice versa (150). Although moment-to-moment changes in spontaneous pain cannot be explored in experimental animal models, the fluctuating nature of the PAG-RVM-SpV pathway observed in this investigation may reflect moment-to-moment changes in the spontaneous activity of On- and Off- cells in the PAG and RVM. Indeed, given that On- and Off- cells show a graded response according to the magnitude of sensory pain behaviours (143), it is likely that fluctuations in RVM cell firing can also contribute to pain intensity in humans.

Furthermore, a recent stimulus-evoked fMRI study explored RVM activity in patients with knee osteoarthritis pain with varying degrees of neuropathic and nociceptive qualities (151). In these patients, the change in RVM BOLD signal during noxious punctate stimulation over the knee was correlated with neuropathic pain scores on the PainDETECT questionnaire. That is, individuals who experienced a neuropathic quality to their pain experienced greater activity within the RVM during noxious punctate stimulation compared to those who experienced more nociceptive pain qualities. Furthermore, the authors reported significantly greater pre-operative RVM BOLD signal in patients who went on to experience moderate-to-severe neuropathic pain 12 months after arthroplasty compared to those who did not (151), possibly reflecting a descending RVM-driven facilitation that maintains pain even after surgical intervention. However, further investigations are required to explore this idea owing to the small sample size in this analysis.

### Locus Coeruleus and SRD Plasticity

In addition to evidence of changes in the PAG-RVM-DH/SpV pathway, there is preclinical evidence that altered functioning of the locus coeruleus and SRD are involved in the development and/or maintenance of chronic neuropathic pain. Due to the vastly different effects of noradrenaline on alpha-1- and alpha-2- adrenoreceptors (91, 94), it has been challenging to ascertain the effects of the locus coeruleus on pain following nerve injury. Some investigations suggest that, following nerve injury, the locus coeruleus continues to inhibit nociception at the DH since the disruption of noradrenaline locally at the spinal cord leads to enhanced mechanical sensitivity after nerve injury (152, 153). In contrast, there is evidence that the locus coeruleus plays an overall pro-nociceptive role following nerve injury and contributes to the maintenance of sensory and affective behavioural responses associated with neuropathic pain (125, 154). For instance, if widespread noradrenergic neurons are selectively destroyed 3 weeks after infraorbital nerve constriction injury, there is a marked reduction in mechanical sensitivity for at least 4 weeks after noradrenergic cell ablation (154). These preclinical studies suggest that neuroplastic changes that compromise the analgesic ability of the locus coeruleus contribute to the pain development (91, 125, 155).

There is also preclinical evidence of pro-nociceptive changes in SRD control over DH firing following nerve injury. In rats with neuropathic pain, blocking SRD activity significantly reduces noxious-evoked and spontaneous activity of spinal WDR neurons (156), which may result from inputs to the SRD from the locus coeruleus (101, 157). Following nerve injury, noxious stimulation increases noradrenaline release in the SRD (101), and blocking this release with a selective viral vector targeting SRD noradrenergic afferents can significantly attenuate allodynia and hyperalgesia (157). These effects are likely not due to changes in tonic drive since preclinical studies indicate that basal locus coeruleus activity levels are unchanged following nerve injury (125, 158). Furthermore, in line with human CPM investigations, numerous preclinical investigations have identified reduced or abolished DNIC following nerve injury (159–161).

**Figure 5:**
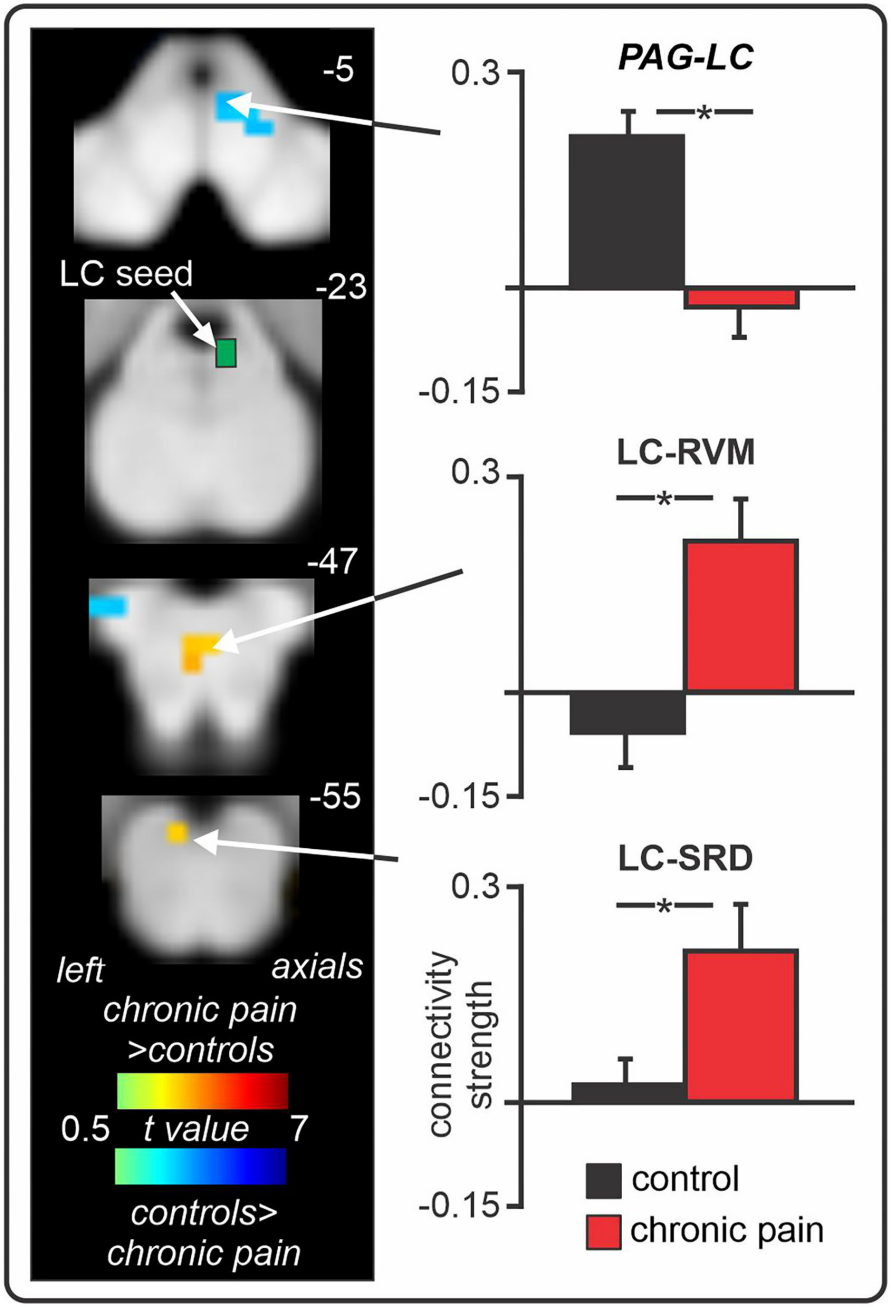
Changes in locus coeruleus (LC) fMRI signal coupling in individuals with chronic orofacial neuropathic pain. Compared to pain-free controls, chronic pain patients show enhanced positive LC signal coupling with the rostral ventromedial medulla (RVM) and subnucleus reticularis dorsalis (SRD). Furthermore, individuals with chronic pain display near-zero signal coupling between the LC and periaqueductal grey (PAG). *significant between-groups difference determined in a voxel-by-voxel analysis. Figure modified with permission from Mills et al. (32).

Consistent with these preclinical findings, in our recent human brain imaging investigation outlined above, we found enhanced resting-state fMRI signal coupling between the locus coeruleus and both the RVM and SRD in chronic orofacial neuropathic pain patients (Figure 5) (32). Altered RVM and LC coupling with the SRD may underlie impaired CPM abilities in these patients, however CPM was not directly tested in this investigation. Interestingly, we found no change in locus coeruleus signal coupling with the SpV, suggesting that a direct descending noradrenergic projection from the locus coeruleus to the DH/SpV may not be involved in the maintenance of ongoing neuropathic pain in humans, in contrast to a recent preclinical finding (154). Of course, this may represent a difference in the role of locus coeruleus-driven noradrenaline at the DH/SpV during evoked-pain behaviours (154) compared to spontaneous pain [as measured by resting-state fMRI (32)]. However, further brain imaging investigations are required to examine the functioning of the locus coeruleus in individuals with other neuropathic pain conditions. Furthermore, we also found reduced (near-zero) connectivity between the locus coeruleus and PAG in patients, suggesting that altered resting interactions between the locus coeruleus and the PAG, SRD, and RVM may collectively contribute to the pro-nociceptive effects and reduced CPM abilities that are often observed in patients with chronic neuropathic pain.

Whilst beyond the scope of this review, there is also evidence that neuropathic pain is associated with plasticity in the connexions between the brainstem circuitry and higher cortical and sub-cortical regions involved in pain processing and modulation. For example, individuals with post-herpetic neuralgia show weaker functional connectivity between the PAG and anterior cingulate cortex than controls (162), suggesting that ongoing recruitment of cortical anti-nociceptive systems may be weakened in these patients. In contrast, stronger functional connectivity between the PAG and the thalamus has been observed in individuals with chronic neuropathic pain (32, 162), possibly reflecting changes in ascending nociceptive pathway activity. Preclinical studies have identified that neuropathic pain is associated with morphological and functional changes in the medial prefrontal cortex, including reduced inhibitory drive from the medial prefrontal cortex to the PAG (163–165). The authors suggest that there is a shift in the excitatory/inhibitory balance in this circuit, which may contribute to a pro-nociceptive shift in PAG descending projections and thus the persistence of ongoing pain following nerve injury, an idea supported by a recent investigation in which mPFC connexions with the DNIC circuitry were found to contribute to reduced DNIC in neuropathic rats (161). Overall, these findings suggest that chronic neuropathic pain is associated with a functional shift in the descending projections from cortical regions to the brainstem pain-modulation circuits. Ultimately, these descending connexions likely also contribute to a pro-nociceptive state in individuals with neuropathic pain.

## Conclusions and Future Directions

Overall, there is growing evidence from preclinical and human studies that there is plasticity in the structure and function of brainstem pain-modulation circuits in chronic neuropathic pain conditions. This evidence suggests that following nervous system injury, altered brainstem pain modulatory circuit control over the DH/SpV results in a pro-nociceptive state, contributing to a reduced analgesic ability during pain-modulation experimental paradigms and an increased sensitivity to incoming somatosensory stimuli. In addition, there is growing evidence that the state of this brainstem system prior to injury is critical for the subsequent development of chronic pain, a situation that may reflect an individual's ability to respond adequately to the cascade of events that occur at the DH/SpV following nerve injury. Whilst evidence for such plasticity is growing, more studies are needed before we can fully understand the role of the brainstem in the initiation and maintenance of chronic neuropathic pain conditions.

Indeed, future studies will benefit from the recent technological advancements to provide a more in-depth exploration of the brainstem circuitry. As alluded to earlier, the development of ultra-high field 7 Tesla MRI now offers a more detailed view of the brainstem nuclei involved in pain-modulation, both in acute and chronic pain states. In particular, future studies could use 7T MRI to explore brainstem structure and function in individuals with different neuropathic pain conditions so that more personalised profiles of pain-modulation system functioning can be determined and more targeted treatments can be developed for different pain conditions. Furthermore, future investigations could also focus on changes in brainstem pain circuitry functioning within individual subjects to a greater extent in order to gain a more detailed understanding of changes in system functioning in individuals with chronic neuropathic pain. A similar approach has been used to study changes in brainstem function in individuals with migraine across the migraine cycle (31, 166). For instance, it would be valuable to study changes in pain-modulation circuitry functioning over the course of an individual's pain development – from immediately following nerve injury throughout the development and persistence of long-term pain, and to explore whether there are changes in circuitry functioning following the administration of a standard pain treatment. Moreover, exploring brainstem function *prior* to planned surgeries would provide crucial evidence to support or refute the idea that the state of the brainstem system prior to injury can influence an individual's propensity for developing chronic pain. Overall, the use of human brain imaging has greatly aided our current understanding of endogenous pain-modulation circuitry functioning in both acute and chronic neuropathic pain states, and will certainly continue to provide invaluable information that will ultimately help us better treat neuropathic pain conditions.

## Author Contributions

EM, KK, and LH contributed to initial design and outline of manuscript. EM wrote initial manuscript draught and then edited all subsequent versions. KK and LH edited all versions of manuscript. All authors contributed to the article and approved the submitted version.

## Conflict of Interest

The authors declare that the research was conducted in the absence of any commercial or financial relationships that could be construed as a potential conflict of interest.

## Publisher's Note

All claims expressed in this article are solely those of the authors and do not necessarily represent those of their affiliated organizations, or those of the publisher, the editors and the reviewers. Any product that may be evaluated in this article, or claim that may be made by its manufacturer, is not guaranteed or endorsed by the publisher.
