# Corrigendum: Brainstem Pain-Modulation Circuitry and Its Plasticity in Neuropathic Pain: Insights From Human Brain Imaging Investigations

**DOI:** 10.3389/fpain.2021.812209

**Published:** 2021-12-21

**Authors:** Emily P. Mills, Kevin A. Keay, Luke A. Henderson

**Affiliations:** Brain and Mind Centre, School of Medical Sciences (Neuroscience), University of Sydney, Sydney, NSW, Australia

**Keywords:** periaqueductal grey, rostral ventromedial medulla, locus coeruleus, subnucleus reticularis dorsalis, chronic neuropathic pain, conditioned pain modulation, functional magnetic resonance imaging, analgesia

In the original article, there was a mistake in the legend for **Figure 4** as published. This legend is not fully cited, and Dove Medical Press requires acknowledgement as the original publisher. The correct legend appears below.

**Figure 4**. Spontaneous changes in periaqueductal grey (PAG) - rostral ventromedial medulla (RVM) - spinal trigeminal nucleus (SpV) fMRI signal coupling in chronic neuropathic orofacial pain. Compared to controls, individuals with chronic pain show enhanced positive functional connectivity between the RVM “seed” and the PAG and SpV, in addition to the locus coeruleus (LC) and subnucleus reticularis dorsalis (SRD). Furthermore, in neuropathic pain patients, as the intensity of their clinical pain changes throughout a 12-min fMRI scan, so too do their RVM connectivity strengths with the PAG and SpV. That is, when pain intensity is spontaneously low, RVM connectivity strengths with both the PAG and SpV are low; and when pain intensity is spontaneously high, RVM connectivity strengths are high and positive. *significant between-groups difference determined in a voxel-by-voxel analysis. Figure modified with permission from (1) and Mills et al. Journal of Pain Research 2020:13:2223–2235; Originally published by and used with permission from Dove Medical Press Ltd. (2).

The authors apologize for this error and state that this does not change the scientific conclusions of the article in any way. The original article has been updated.

## Publisher's Note

All claims expressed in this article are solely those of the authors and do not necessarily represent those of their affiliated organizations, or those of the publisher, the editors and the reviewers. Any product that may be evaluated in this article, or claim that may be made by its manufacturer, is not guaranteed or endorsed by the publisher.
